# Septins Regulate Bacterial Entry into Host Cells

**DOI:** 10.1371/journal.pone.0004196

**Published:** 2009-01-15

**Authors:** Serge Mostowy, To Nam Tham, Anne Danckaert, Stéphanie Guadagnini, Stéphanie Boisson-Dupuis, Javier Pizarro-Cerdá, Pascale Cossart

**Affiliations:** 1 Institut Pasteur, Unité des Interactions Bactéries-Cellules, Paris, France; 2 Inserm, U604, Paris, France; 3 INRA, USC2020, Paris, France; 4 Institut Pasteur, Plate-forme d'Imagerie Dynamique, Paris, France; 5 Institut Pasteur, Plateforme de Microscopie Electronique, Paris, France; Duke University Medical Center, United States of America

## Abstract

**Background:**

Septins are conserved GTPases that form filaments and are required in many organisms for several processes including cytokinesis. We previously identified SEPT9 associated with phagosomes containing latex beads coated with the *Listeria* surface protein InlB.

**Methodology/Principal Findings:**

Here, we investigated septin function during entry of invasive bacteria in non-phagocytic mammalian cells. We found that SEPT9, and its interacting partners SEPT2 and SEPT11, are recruited as collars next to actin at the site of entry of *Listeria* and *Shigella*. SEPT2-depletion by siRNA decreased bacterial invasion, suggesting that septins have roles during particle entry. Incubating cells with InlB-coated beads confirmed an essential role for SEPT2. Moreover, SEPT2-depletion impaired InlB-mediated stimulation of Met-dependent signaling as shown by FRET.

**Conclusions/Significance:**

Together these findings highlight novel roles for SEPT2, and distinguish the roles of septin and actin in bacterial entry.

## Introduction

Septins were originally identified in *Saccharomyces cerevisiae* as required for septum formation and cell division [1]. Septins are small GTPases of 30–65 kDa found in fungi and animals sharing at least a conserved role in cytokinesis [2], [3]. Mammalian septins are increasingly recognized for other cellular functions including roles in cell membrane dynamics [4], [5], vesicle fusion events [6], [7], and the assembly of signaling complexes beneath the plasma membrane [8]. Septin dysfunction has been associated with several human pathological conditions such as cancer [9], hereditary neuralgic amyotrophy [10], and Parkinson disease [11]. Despite these important implications, the molecular functions of septins are poorly understood.

Fourteen human septin genes have been identified, and septins are classified on the basis of sequence identity into 4 groups consisting of the SEPT2 group (also called group 2B: SEPT1, SEPT2, SEPT4, SEPT5), the SEPT3 group (also called group 1A: SEPT3, SEPT9, SEPT12), the SEPT6 group (also called group 1B: SEPT6, SEPT8, SEPT10, SEPT11, SEPT14), and the SEPT7 group (a subclassification of group 2B: SEPT7, SEPT13) [2], [12]. Septins polymerize into hetero-oligomeric protein complexes that form filaments, and associate with cellular membranes, actin filaments, and microtubules [8], [13]. Thus, septins are increasingly regarded as a novel cytoskeletal component. In vitro, bundles of septin filaments can form rings of approximately 0.6 µm in diameter [14]. The crystal structure of a septin complex (SEPT2-SEPT6-SEPT7) was recently solved [15]. The structure demonstrated a central role for SEPT2 in filament formation, and definitively established that septins, as opposed to actin and microtubules, form non-polar filaments. Despite these and other structural insights [12], [16], to what degree septins represent a distinct component of the cytoskeleton remains to be determined. Furthermore, the extent to which other septin complexes, such as SEPT7-SEPT9-SEPT11 [17] or SEPT5-SEPT7-SEPT11 [18], share properties and/or functionally compete with the crystallized SEPT2-SEPT6-SEPT7 complex is not yet known.

*Listeria monocytogenes* is an intracellular pathogen able to induce its own internalization into non-phagocytic cells [19]. Of the several bacterial surface proteins that participate in *Listeria* entry into non-phagocytic cells [20], the two best characterized are InlA and InlB, either of which is independently sufficient to promote bacterial internalization into mammalian epithelial cells that express its respective receptor. InlA interacts with the surface protein E-cadherin [21], while InlB recognizes the receptor tyrosine kinase Met [22]. These different interactions both have well-described cell signaling consequences, including actin polymerization and membrane extension, which ultimately lead to bacterial uptake [23], [24]. In order to identify other cellular effectors involved in the early steps of *Listeria* infection, we previously performed a proteomic analysis of phagosomes containing latex beads coated with InlA or InlB [25]. This approach allowed us to detect SEPT9 associated with InlB-bead phagosomes, implicating septins for the first time in bacterial infection.

In contrast to the well-established roles of actin [26], [27] and microtubules [28], [29] during the infection process, septin function during microbial invasion has not yet been investigated. We thus decided to address this issue. We first show that SEPT9 interacts with several septins, including the core SEPT2. By infecting human cell lines we show septin recruitment at the site of bacterial entry. We then use siRNA and demonstrate a role for SEPT2 in the bacterial entry process.

## Results

### SEPT9 interacts with septins from other groups

A hallmark of septins is their ability to form protein complexes and filaments. Characterized complexes isolated from mammalian cells include SEPT2-SEPT6-SEPT7 [14], [15] and SEPT7-SEPT9-SEPT11 [17]. We previously found SEPT9 in association with phagosomes containing InlB-coated beads [25]. To identify unknown molecules interacting with SEPT9, we performed yeast-two hybrid screens using SEPT9 as bait against human placental and colon cDNA libraries, and identified SEPT2, SEPT6, SEPT7, and SEPT11 as binding partners of SEPT9.

**Figure 1:**
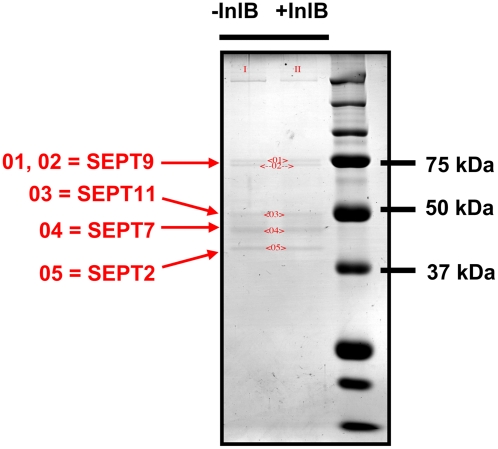
Binding partners of SEPT9. To search for binding partners of SEPT9, we performed immunoprecipitation experiments using antibodies for SEPT9 and lysates from untreated HeLa cells (−InlB), or HeLa cells stimulated for 2 minutes with 5 nM (total) of purified InlB (+InlB). Mass spectrometry was subsequently performed on samples analyzed by SDS-PAGE, where in both cases SEPT2, SEPT7, and SEPT11 were identified to coimmunoprecipitate with SEPT9.

We also used immunoprecipitation in HeLa cells, a cell line that expresses the InlB receptor but not the InlA receptor. Using antibodies for SEPT9, we performed immunoprecipitation experiments on cells stimulated or not with purified InlB, and identified by mass spectrometry, in both cases, SEPT2, SEPT7, and SEPT11 coimmunoprecipitating with SEPT9 (Figure 1).

Together these data indicate that SEPT9 forms higher order structures with other septins in vivo. SEPT2, SEPT7, and SEPT6/SEPT11 each represent a different septin group, suggesting that SEPT9 interacts with members of other groups to form a complex, and/or participates in the formation of previously unidentified septin complexes.

### Expression, localization, and recruitment during bacterial invasion of SEPT2, SEPT9, and SEPT11 in human non-phagocytic cells

**Figure 2:**
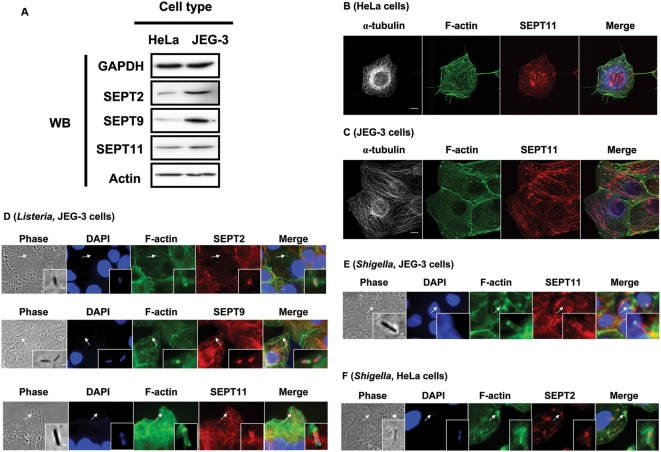
Septin expression, localization, and recruitment at the bacterial entry site in human non-phagocytic cells. (A) Western blots (WB) of septins in non-phagocytic cell lines. HeLa and JEG-3 cells were harvested, and cell lysates were separated by 10% SDS-PAGE before immunoblotting. Blots were probed with antibodies specific to GAPDH, SEPT2, SEPT9, SEPT11, and actin. Blots for GAPDH are shown as a loading control. (B) Septin filaments partially colocalize with actin filaments in HeLa cells. Endogenous α-tubulin, F-actin, and SEPT2, SEPT9, or SEPT11 were visualized by immunostaining with anti-α-tubulin (grey and in Merge blue), anti-F-actin (green), and anti-SEPT2, anti-SEPT9, or anti-SEPT11 antibodies (red). Representative confocal images for α-tubulin, F-actin, and SEPT11 distribution in HeLa cells are here displayed, and similar images were obtained labeling for SEPT2 or SEPT9. Scale bars indicate 10 µm. (C) Septin filaments partially colocalize with microtubules in JEG-3 cells. Endogenous α–tubulin, F-actin, and SEPT2, SEPT9, or SEPT11 were visualized for immunostaining with anti-α-tubulin (grey and in Merge blue), anti-F-actin (green), and anti-SEPT2, anti-SEPT9, or anti-SEPT11 antibodies (red). Representative confocal images for α-tubulin, F-actin, and SEPT11 distribution in JEG-3 cells are here displayed, and similar images were obtained labeling for SEPT2 or SEPT9. Scale bars indicate 10 µm. (D) Septin recruitment at the site of *Listeria* entry in JEG-3 cells. Cells were infected with *L. monocytogenes* BUG 1641 for 5, 10, or 15 minutes and then fixed for microscopy. Endogenous septin was stained with anti-SEPT2, anti-SEPT9, or anti-SEPT11 antibodies (red). Actin was stained with anti-F-actin (green), and *Listeria* was marked using DAPI (blue). Representative photos are here displayed, where inset images highlight the septin collar-like recruitment around *Listeria* to which the white arrows are pointing. Scale bars indicate 1 µm. (E, F) Septin recruitment to the site of *Shigella* entry in (E) JEG-3 cells or (F) HeLa cells. Cells were infected with *S. flexneri* M90T for 15 minutes and then fixed for microscopy. Endogenous septin was stained with anti-SEPT2, anti-SEPT9, or anti-SEPT11 antibodies (red). Actin was stained with anti-F-actin (green), and *Shigella* was marked using DAPI (blue). Representative photos showing SEPT11 (JEG-3 cells) and SEPT2 (HeLa cells) are here displayed, where inset images highlight the septin collar-like recruitment around *Shigella* to which the white arrows are pointing. In JEG-3 cell and HeLa cells, similar recruitment was obtained when labeling for SEPT2, SEPT9, or SEPT11. Scale bars indicate 1 µm.

Before starting to analyze if septin proteins play a role in *Listeria* invasion of non-phagocytic cells, we first compared by Western blotting septin expression in two different human epithelial cell lines: the well-studied HeLa cells, and JEG-3 cells, a trophoblast cell line that expresses both the InlA and InlB receptors. As our antibodies against SEPT2, SEPT9, and SEPT11 could be used in both Western blotting and immunofluorescence labeling, we focused our analysis on these septins. Figure 2A shows that SEPT2, SEPT9, and SEPT11 were more expressed in JEG-3 cells as compared to HeLa cells. We then confirmed using immunofluorescence labeling that SEPT2, SEPT9, and SEPT11 form filaments that partially colocalize with actin filaments and microtubules in non-dividing, non-infected HeLa and JEG-3 cells (Figure 2B and C) [8], [13].

To determine whether septins are involved in *Listeria* entry, a process known to involve actin [30], [31], we looked for septin recruitment during bacterial invasion. SEPT2, SEPT9, and SEPT11 were abundantly recruited with actin in JEG-3 cells (Figure 2D). Strikingly, septins did not strictly colocalize with actin, and often displayed a collar shape next to actin which is known to be enriched at the pole of invading bacteria [27]. To visualize recruitment in real-time, we co-transfected JEG-3 cells with actin-YFP and SEPT9-CFP, and observed cells by videomicroscopy during the early steps of *Listeria* invasion. Videomicroscopy confirmed septin collar-like recruitment at the site of *Listeria* entry (Movie S1). We also looked for septin accumulation during invasion of *Shigella flexneri*, a pathogen that enters host cells in a different manner than *Listeria*, i.e. via the type III secretion system [32]. In JEG-3 cells, septins were also recruited at the site of *Shigella* entry and formed rings around recently internalized bacteria (Figure 2E). It is interesting to note that the widths of *Listeria* and *Shigella* approximately range from 0.4 to 0.5 µm, consistent with the inner diameter of ring formation of recombinant septin complexes in vitro [14].

We then analyzed septin recruitment during bacterial invasion in HeLa cells. Septin collars could not be consistently observed at sites of *Listeria* entry in HeLa cells as observed in JEG-3 cells. We thus quantified the percentage of actin-positive *Listeria* that also recruit septin during entry. In JEG-3 cells, 75% of actin-positive *Listeria* had collars of SEPT2, SEPT9, or SEPT11. In HeLa cells, 8% of actin-positive *Listeria* recruited discernable septin collars. Our interpretation is that septin collars are also present with actin at the site of bacterial entry into HeLa cells, but in quantities too low to be systematically detectable. In the case of *Shigella* entry in HeLa cells, septin collars were also observed (Figure 2F).

### SEPT2 inactivation decreases bacterial invasion

**Figure 3:**
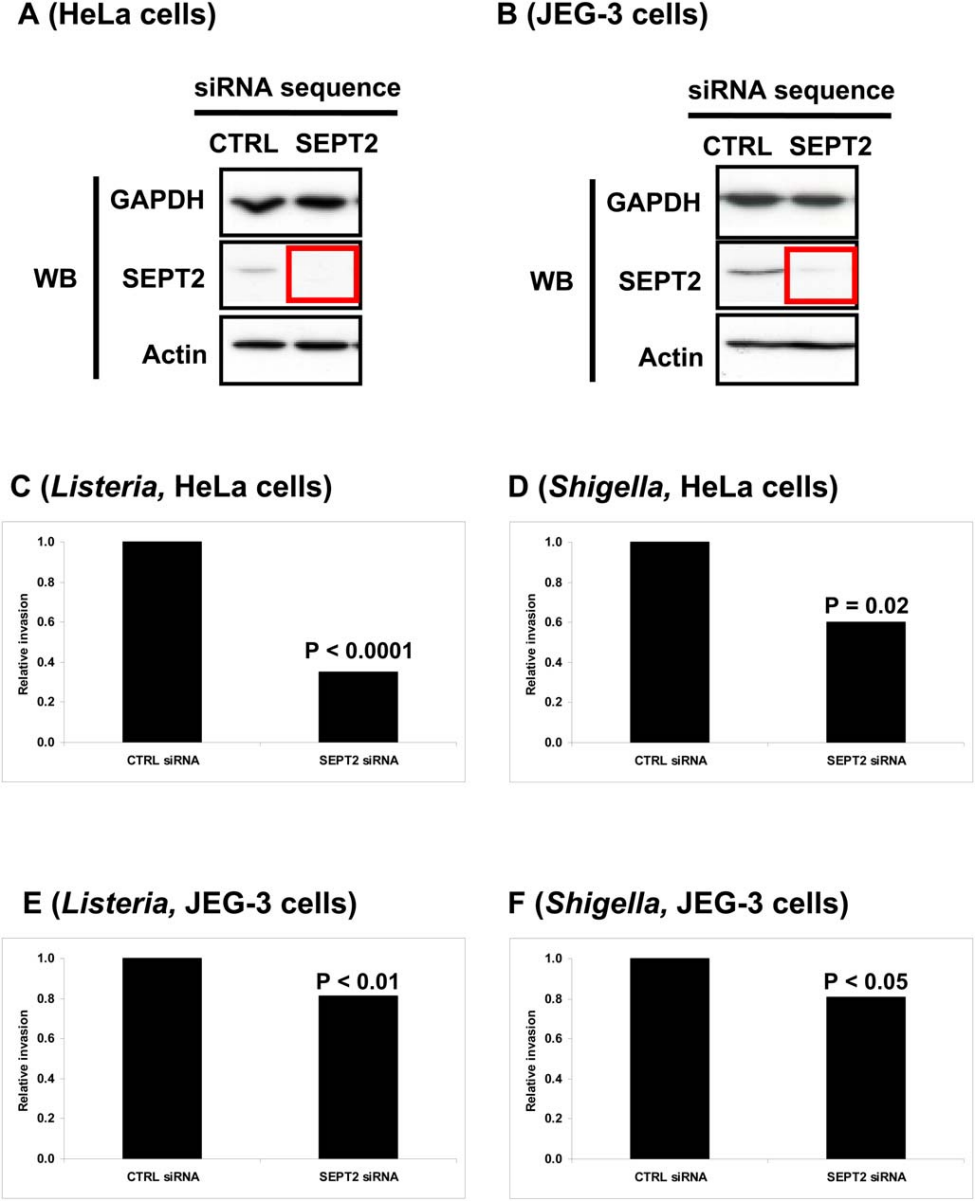
The impact of SEPT2-depletion in HeLa and JEG-3 cells on bacterial entry. (A, B) Western blot (WB) of (A) HeLa cells or (B) JEG-3 cells transfected with siRNA against control (CTRL) or SEPT2. Cell lysates were separated by 10% SDS-PAGE before immunoblotting. The blots were probed with antibodies specific to GAPDH, SEPT2, and actin. GAPDH is shown as a loading control. The red box outlines depleted protein levels for SEPT2. (C–F) SEPT2 regulates the invasion of *L. monocytogenes* and *S. flexneri*. Gentamicin survival assays for (C and E) *L. monocytogenes* EGD or (D and F) *S. flexneri* M90T were performed in (C and D) HeLa cells or (E and F) JEG-3 cells treated with control (CTRL) siRNA, or siRNA targeted against SEPT2. Graphs represent the relative number of intracellular bacteria found inside siRNA-treated cells after the survival assay, where CFU counts obtained from septin-depleted cells were normalized to CTRL siRNA-treated cells. On the graph CTRL siRNA is figuratively presented as 1, and data represents the mean from n ≥9 experiments. Results were analyzed for statistical significance using the Student's t-test.

HeLa cells, which we routinely use for siRNA depletion experiments, and JEG-3 cells were treated with siRNA against SEPT2, the septin protein central to complex and filament formation [15]. siRNA depletion of SEPT2 protein was verified by Western blot (Figure 3A and B). Expression levels of actin were not affected.

To assess the functional consequence of septin-depletion on bacterial entry, we employed the classical gentamicin survival assay [33]. In this assay, internalized bacteria are plated and counted 24 hours after host cell lysis. Colony counts revealed that SEPT2 siRNA treatment significantly decreased *Listeria* and *Shigella* invasion into HeLa cells (P<0.0001 and P = 0.02 respectively) (Figure 3C and D) and JEG-3 cells (P<0.01 and P<0.05 respectively) (Figure 3E and F), revealing a role for SEPT2 during bacterial entry.

### SEPT2 inactivation specifically modulates InlB-mediated entry

Latex beads coated with purified recombinant InlA or InlB protein have been extensively used to study the InlA- and InlB- entry pathways into mammalian cells [34], [35]. To understand further why *L. monocytogenes* invasion is decreased in HeLa cells upon SEPT2-depletion, we investigated particle uptake using 1 µm InlB-coated latex beads. Note that HeLa cells do not express E-cadherin, the host receptor for InlA.

**Figure 4:**
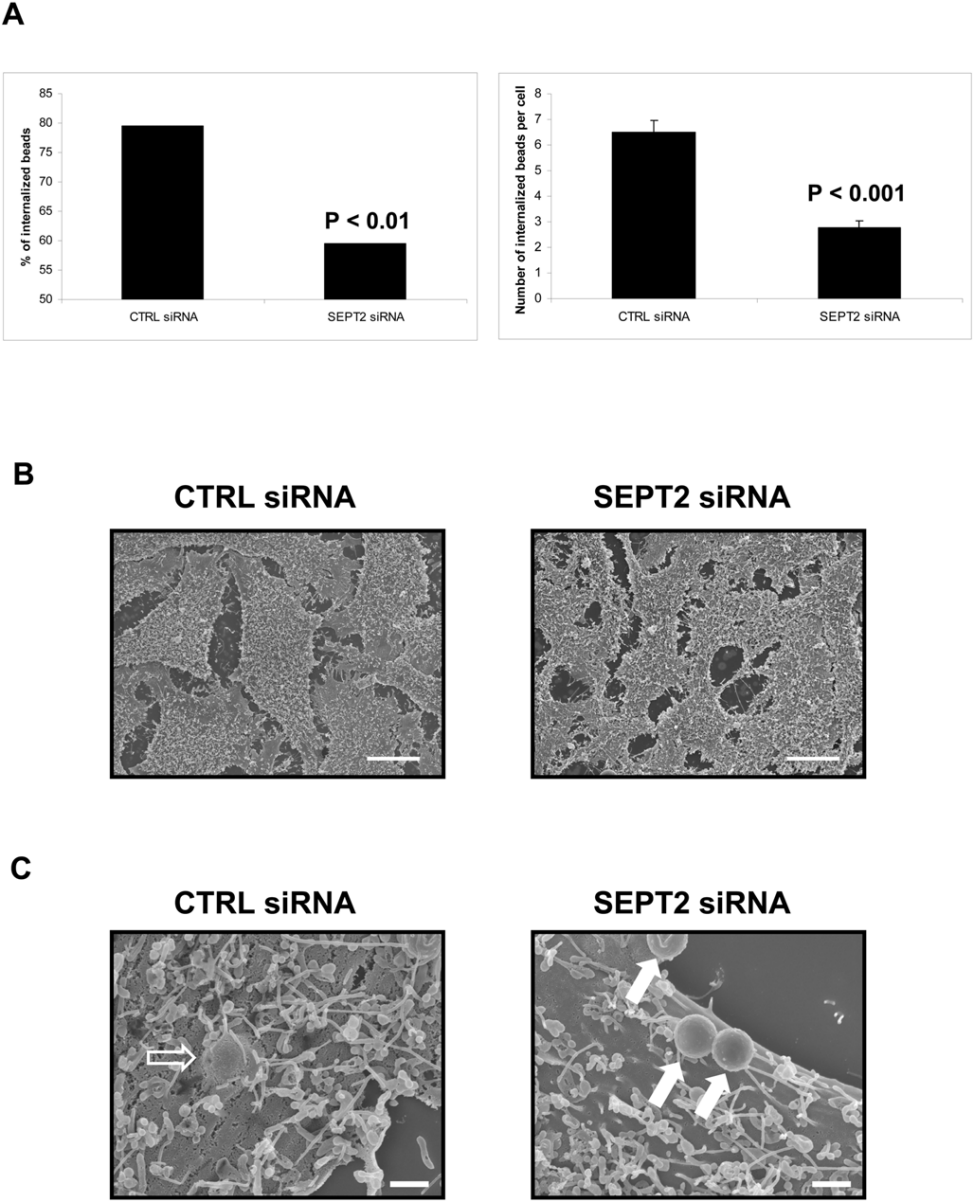
SEPT2 inactivation impairs the entry of InlB-coated beads. (A) Quantification of InlB-induced phagocytosis in control (CTRL) and SEPT2-depleted HeLa cells. For internalization assays, beads were analyzed by immunofluorescence (see Materials and Methods) for being extracellular or intracellular in at least 50 host cells counted for each of n≥2 separate experiments per siRNA treatment. The left graph depicts the total % of internalized 1 µm InlB-coated beads at 5 minutes post-incubation of siRNA-treated cells, calculated from the (total number of internalized beads) / (total number of cell-associated beads) ×100. Results were analyzed for statistical significance using the z-test for percentages. The right graph depicts the average number of internalized 1 µm InlB-coated beads per cell at 5 minutes post-incubation of siRNA-treated cells. Graphed data represent this average value ±SEM, where results were analyzed for statistical significance using the two sample z-test. (B) Representative scanning electron microscopy images of siRNA-treated HeLa cells to depict the membrane surface of control (CTRL) and SEPT2-depleted cells. Magnification = 1500×, where scale bars indicate 10 µm. (C) Uptake of InlB-beads by siRNA-treated cells. Control (CTRL) and SEPT2-depleted HeLa cells were incubated with 1 µm InlB-beads for 5 minutes, and cells were processed for scanning electron microscopy. Depicted here are representative images of InlB-beads internalized (i.e. CTRL cells), or only adherent (i.e. SEPT2-depleted cells), for siRNA treatments. Hollow arrows indicate internalized beads, solid white arrows indicate extracellular adherent beads. Magnification = 15000×, where scale bars indicate 1 µm.

siRNA-treated cells were incubated with InlB-beads, and were examined by a double immunofluorescence microscopy technique in order to distinguish intra- versus extracellular beads and provide a measure of InlB-induced phagocytosis [30]. At 5 minutes post-inoculation, bead internalization was severely impaired in SEPT2-depleted cells compared to control cells (P<0.01 and P<0.001) (Figure 4A).

Scanning electron microscopy was also used to examine the uptake of InlB-beads (Figure 4B and C). Of note, the membrane surface of SEPT2-depleted cells presented microvilli-like structures as do control cells. At 5 minutes post-incubation, the majority of beads associated with control cells were already internalized, while remaining beads were adherent to the cell surface presumably engaged in initial stages of the uptake process. In contrast, beads were less frequently internalized in SEPT2-depleted cells. Together, these data definitively establish a role for SEPT2 during the InlB-mediated internalization process.

### SEPT2 has a critical role in signaling events in response to Met stimulation by InlB

**Figure 5:**
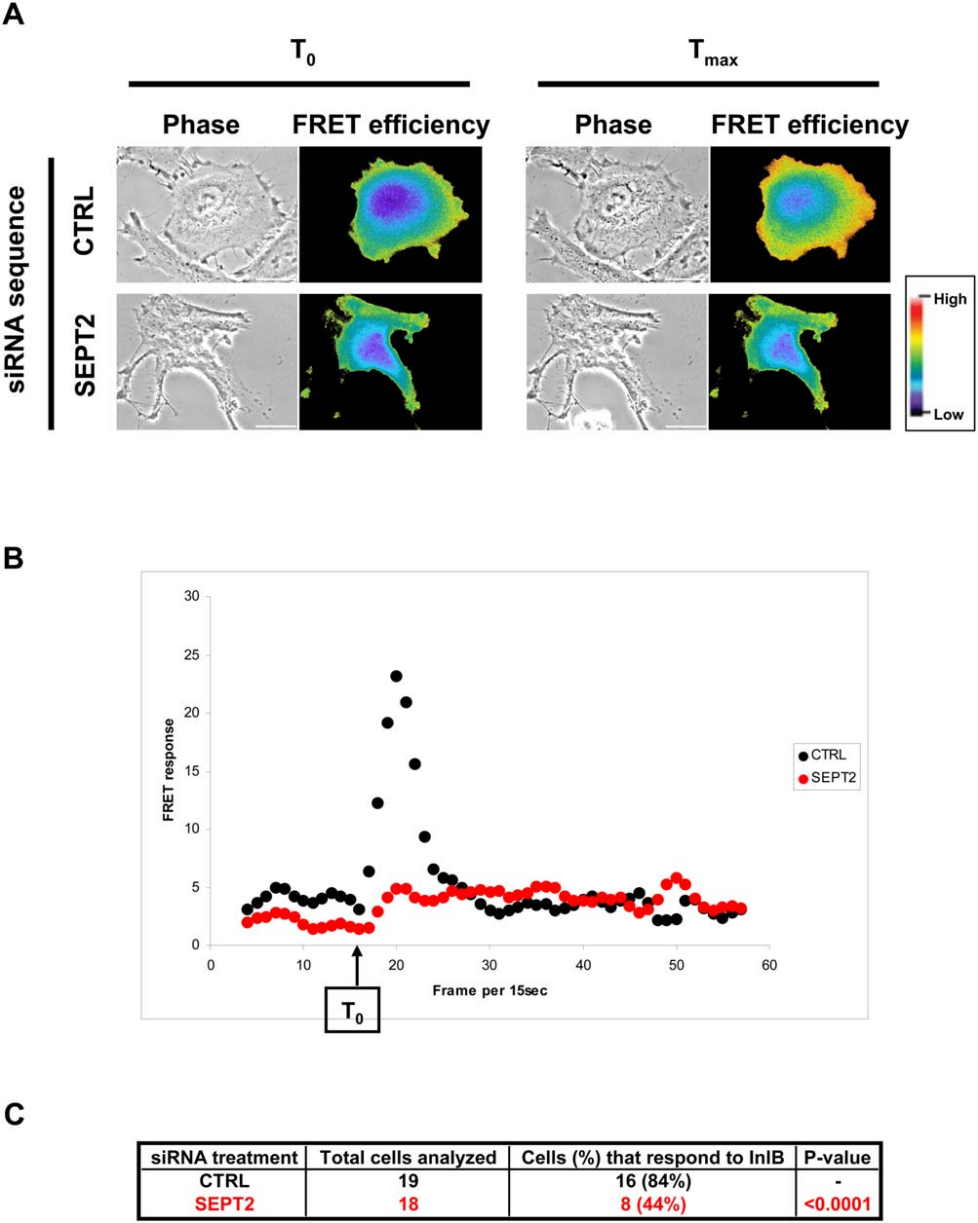
SEPT2-depleted cells respond less to InlB stimulation. (A) Representative phase and FRET efficiency images of control (CTRL) and SEPT2-depleted HeLa cells expressing YFP-AktPH and CFP-AktPH stimulated with InlB. FRET efficiency at 2 time points are presented for each siRNA-treatment: T_0_ (frame 16) and T_max_ (frame corresponding to the maximum induction of FRET efficiency) after stimulation. Pseudocolor scale represents the range of FRET efficiency values from original signal ranging from blue (low) to red (high). Scale bars indicate 10 µm. Movies for the entire timecourse of InlB stimulation for these cells can be observed as Movies S2 and S3. (B) Quantification of FRET response in siRNA-treated cells. Processed FRET response was plotted against time (i.e. frame per 15 seconds), for the representative siRNA-treated cells depicted in Figure 5A. Values for the control (CTRL) cell are plotted in black, and the SEPT2-depleted cell in red. (C) Percentage of cells that respond to InlB treatment. Processed FRET response was determined for 18 or more experiments for each siRNA treatment. Cells were classified as not responding to InlB stimulation if the slope of processed FRET response could not be distinguished above baseline values (i.e. the SEPT2-depleted cell in Figure 5B). The percentage of SEPT2-depleted cells classified as not responding to InlB stimulation was statistically compared to expectations as derived from control cells by Chi-Square test.

*Listeria* enter HeLa cells via interactions between the cell surface receptor Met and the bacterial ligand InlB. Given the impact of SEPT2 depletion on particle entry, we hypothesized that signaling events induced upon InlB/Met interaction should be affected. We focused our study on one signaling pathway induced upon InlB/Met interaction, that of phosphoinositide (PI) 3-kinase activation, and used a fluorescence resonance energy transfer (FRET)-based approach previously developed in our lab [36]. The assay is based on the fact that 3′-phosphoinositides produced by PI 3-kinase upon InlB-induced Met activation recruit the serine/threonine kinase Akt from the cytosol to the plasma membrane. SEPT2-depleted cells and control cells were co-transfected with plasmids encoding YFP-AktPH and CFP-AktPH. Morphological alterations were used as a gauge of which cells were efficiently silenced for SEPT2. The FRET stoichiometry measurement between YFP-AktPH and CFP-AktPH was then performed to determine FRET efficiency (Figure 5A and B, and Movie S2 and S3). As shown in Figure 5C, cells depleted of SEPT2 responded significantly less to InlB stimulation (P<0.0001), highlighting that SEPT2 has a critical role in signaling events contributing to Akt recruitment, i.e. PI 3-kinase activation during InlB/Met signaling.

## Discussion

Septins are small GTPases that can form filaments, and are increasingly recognized as new cytoskeleton components different from actin, microtubules, and intermediate filaments [12], [13]. SEPT9 was previously identified by our laboratory to associate with InlB-bead phagosomes in non-phagocytic cells [25]. We demonstrate here that during infection septins and actin can be detected at the bacterial entry site, though septins form striking collar shape structures around entering bacteria similar to those found at the yeast mother-bud neck. Using siRNA to specifically establish a role for septins, we reveal that SEPT2 is critical for the bacterial entry process. This strongly introduces septins as new regulators of bacterial entry and phagocytosis, and highlights that septins have previously unrecognized cellular functions.

### SEPT2 regulates the InlB/Met-mediated entry of Listeria into host cells

We have here performed our functional analysis on HeLa cells, a cell line that does not express E-cadherin and thus allows analysis of the *Listeria* InlB/Met entry pathway [24]. We could reveal that depletion of SEPT2 decreased the InlB/Met-mediated entry of *L. monocytogenes*. Furthermore, FRET analysis showed that SEPT2 is required for the appropriate signaling cascade in response to Met stimulation by InlB. Taken together, our data reveal an effect of SEPT2-depletion on the interaction of InlB with its host receptor Met, and consequently on bacterial entry.

Recent structural data has demonstrated that septins are ordered in the isolated complex as SEPT7-SEPT6-SEPT2-SEPT2-SEPT6-SEPT7 [15]. As the central subunit in this septin complex, SEPT2 has so far been described as not ‘replaceable’ by other septins. All our findings, starting from the observation that SEPT2-depletion consistently has a negative impact on bacterial invasion (Figures 3C–F), would argue that SEPT2 is indeed not replaceable at least in the cell types tested.

### Septins form collars at the site of bacterial entry as at the yeast mother-bud neck

During *L. monocytogenes* entry in host cells (Figure 2D), actin is enriched at the invading pole of the bacteria, while septins form ring structures next to the actin-enriched region around incompletely-internalized bacteria in a geometry resembling ring structures observed at the yeast mother-bud neck [8], [12]. Septin rings are similarly detected at the site of entry of *Listeria innocua* expressing InlA and consequently capable of entering host cells via E-cadherin [37], and *Escherichia coli* expressing the *Yersinia* outer membrane protein invasin and consequently capable of entering host cells via β1-integrin [38] (data not shown). Moreover, septin rings are also detected at the site of entry of *S. flexneri* (Figure 2E and F) and *Salmonella* Typhimurium (data not shown), suggesting that septin recruitment is a general occurrence wherever there is actin polymerization upon bacterial entry. These results have been observed in several mammalian non-phagocytic cell lines (our unpublished results), and are in agreement with recent work showing that septin recruitment is required for phagosome formation during FcγR-mediated phagocytosis in macrophages [4]. However, we show here characteristic differences in the architecture of actin and septin enrichment at the site of bacterial entry, strongly suggesting distinct and probably complementary roles for actin and septin in the process of entry. Whether or not septin filaments assemble directly at the site of entry is currently unknown, and future study of bacterial invasion will address the kinetics and function of septin ring formation.

The mechanisms underlying septin recruitment at the site of particle entry, and more generally how septins interact with membranes, is not yet clear. To begin to investigate the signals responsible for septin accumulation at the site of bacterial entry, JEG-3 cells were treated with cytochalasin D which inhibits actin polymerization. This treatment resulted in an inhibition of both actin and septin accumulation at the site of *Listeria* entry (data not shown). Accumulation of septins at the phagocytic cup is thus intimately linked to the accumulation of actin, albeit in an unknown fashion. In contrast, inhibition of microtubule polymerization by pretreatment of JEG-3 cells with nocodazole did not prevent actin and septin accumulation at the site of *Listeria* or *Shigella* entry (data not shown).

Septins are capable of binding to phosphatidylinositol lipids [39], and thus could be targeted to the *Listeria* entry site by these secondary messengers. Considering that PI 4-kinase type II and PI 3-kinase type I are required for *Listeria* internalization of target cells [40], it is possible that these, and/or other lipid kinases, play a role in the spatial and temporal recruitment of septins during bacterial entry. In agreement with this proposal, during FcγR-mediated phagocytosis of latex beads, septin accumulation was colocalized with the phosphatidylinositol lipid PIP_2_ [4].

### Do septins interact with other proteins during bacterial entry?

Activation of PI 3-kinase by InlB in HeLa cells leads to the activation of the small Rho GTPases Rac1 and Cdc42, which act upstream of an elaborate signaling cascade that includes WAVE, N-WASP, LIM-kinase, cofilin, and the Arp2/3 complex [23]. All of these molecules participate in tightly regulated steps of actin polymerization and depolymerization required for *Listeria* uptake [30], [31]. It is now important to determine whether septins play a role in this pathway, or if activation of this pathway regulates septin recruitment. There is evidence that septin filament organization depends on Rho-family GTPase signaling pathways [41], and the organization of septin and actin filaments has been associated to Rhotekin, a downstream effector of Rho signaling [42]. Borg proteins, first identified as effectors of Cdc42 [43], have also been shown to regulate septin organization [44]. How the Rho/Rhotekin, Cdc42/Borgs, and likely other signaling pathways could influence septin assembly to regulate the efficiency of bacterial entry is not yet understood. This could occur potentially by recruiting multi-protein complexes to forming phagosomes on the plasma membrane [4], and/or by contributing towards the spatial organization of actin and/or surface receptors during this actin-based process.

Our findings for the first time highlight a role for a septin in regulating bacterial invasion. Future work will focus on understanding septin function and regulation during not only bacterial entry but also other stages of the infectious life cycle, in particular actin-based movements where septin accumulation has also been observed (data not shown). Ultimately, our goal is to characterize septin function and the contribution of septins as unique cytoskeletal components.

## Materials and Methods

### Mammalian cells, bacterial strains, and culture conditions

HeLa human cervix carcinoma cells (ATCC CCL-2) and JEG-3 human placenta choriocarcinoma cells (ATCC HTB-36) were cultured as recommended in DMEM+GlutaMAX (GIBCO) supplemented with 1% sodium pyruvate (GIBCO) and 10% FCS. Cells were grown at 37°C in 10% CO_2_ atmosphere.

*L. monocytogenes* (strain EGD BUG 600) was grown overnight at 37°C in brain heart infusion (BHI) media (Difco Laboratories), diluted 15× in fresh BHI, and cultured until OD_600 nm_ = 0.8. *L. monocytogenes* BUG 1641 (a hyperinvasive strain expressing an InlB derivative containing the NH_2_-terminal region sufficient for entry which is covalently anchored at the bacterial surface [30]) and *L. innocua* expressing InlA (BUG 1489) [37] was similarly grown, except that media contained 5 µg/ml of erythromycin or chloramphenicol, respectively. *Shigella flexneri* strain M90T (gift from Phillipe Sansonetti, BUG 2505) was cultured in trypticase soy (TCS) as previously described [45]. After being grown overnight, *S. flexneri* was diluted 80× in fresh TCS, and cultured until OD_600 nm_ = 0.6. GFP-expressing *Salmonella enterica* serovar Typhimurium strain 12023 (gift from Stéphane Méresse, BUG 2562) was cultured in Luria Bertani (LB) medium as described in [57]. *Escherichia coli* expressing the *Yersinia* outer membrane protein invasin (gift from Guy Tran Van Nhieu, BUG 2563) was grown in LB medium supplemented with chloramphenicol (35 µg/ml). Before use, bacteria were incubated overnight at 37°C with shaking, diluted 100× in fresh media and incubated in the same conditions for 3.5 hours.

### Gentamicin survival assays

Gentamicin survival assays were performed as previously described [46]. Cells were incubated in DMEM with *Listeria* at an multiplicity of infection (MOI) of 50 for 1 hour at 37°C and 10% CO_2_, washed with DMEM without antibiotic, and subsequently incubated with fresh gentamicin-containing complete media (10 µg/ml) for an additional 1 hour. Cells were washed and then lysed with distilled H_2_O. The number of viable bacteria released from the cells was assessed by plating on BHI agar plates. Each experiment was done in triplicate, and triplicates were performed 3 or more times independently. Infections of cells with *S. flexneri* were performed as previously described [XPATH ERROR: unknown variable "start".], . *Shigella* were added to cells incubated in DMEM at MOI of 100 and centrifuged at 700 g for 10 minutes at 21°C. Cells were then placed at 37°C and 10% CO_2_ for 30 minutes, washed with DMEM without antibiotic, and subsequently incubated for 1 hour in presence of gentamicin (50 µg/ml). Cells were washed twice and then lysed with distilled H_2_O. Lysates were plated for bacterial counting on LB plates.

### Antibodies and reagents

Primary antibodies used in this study included rabbit polyclonals anti-SEPT9 (R69) [25], anti-SEPT2 (gift from William Trimble), and anti-SEPT11 (gift from Makoto Kinoshita). Mouse monoclonal anti-InlB has been described previously [35]. Mouse monoclonal anti-α-tubulin was purchased from Molecular Probes. Mouse monoclonal anti-GAPDH (glyceraldehyde-3-phosphate dehydrogenase), used as a control throughout Western blot experimentation [48], was purchased from AbCam (6C5).

Secondary antibodies used were Cy5-conjugated (Jackson ImmunoResearch Laboratories), Alexa 488–conjugated, and Alexa 546–conjugated goat anti–rabbit and goat anti–mouse antibodies (Molecular Probes). F-actin was labeled with Alexa 488 (FITC)- and Alexa 546-phalloidin (Molecular Probes). For immunoblotting, total cellular extracts were blotted with above-mentioned antibodies, followed by peroxidase-conjugated goat anti-mouse and anti-rabbit antibodies (Biosys Laboratories). Proteins were run on 10% acrylamide gels.

### Yeast-two hybrid screens and immunoprecipitation studies

Yeast-two hybrid screens using DNA sequence of human SEPT9 as bait against human placental and colon cDNA libraries were performed at Hybrigenics (http://www.hybrigenics.com/) as described in [49]. Immunoprecipitations were performed as described in [49], where we probed lysates from HeLa cells (untreated or stimulated for 2 minutes with 5 nM total of purified InlB) looking for binding partners of SEPT9. Cells were lysed in NP-40 buffer (20 mM Tris pH 8.0, 1% (v/v) NP-40, 137 mM NaCl, 10% (v/v) glycerol and protease inhibitors cocktail). Mass spectrometry was performed on samples analyzed by SDS-PAGE at Pasteur-Genopole® Ile-de-France / Plate-forme de Proteomique. The resulting sequences were used to identify the corresponding genes in the GenBank database (NCBI) using a fully automated procedure.

### Preparation of InlB-coated beads

Coating of latex beads with purified InlB and incubation of host cells with InlB-coated latex beads were performed as previously described [25], [50], where 1 µm diameter latex beads were coated following the manufacturer's instructions (Molecular Probes). Prior to addition, InlB-coated beads were briefly sonicated, diluted in DMEM, and added to siRNA-treated HeLa cells at a multiplicity of approximately 100 beads per cell.

For kinetic analysis by immunofluorescence and scanning electron microscopy, host cells were stored at 4°C for 5 minutes before incubation with beads, then beads diluted in DMEM were added and cells were centrifuged at 1000 g for 1 minute at 4°C. Medium was removed and replaced with fresh DMEM. Cells were then incubated at 37°C and 10% CO_2_ for 5 minutes, after which they were washed twice with DMEM, and processed for immunofluorescence or scanning electron microscopy analysis.

### Immunofluorescence and confocal microscopy

Immunofluorescence analysis was performed as previously described [46]. Briefly, 10^5^–10^6^ HeLa or JEG-3 cells were plated onto glass coverslips in 6-well plates (TPP), and used for experiments 48 hours later. Cells on coverslips were fixed 15 minutes in 4% PFA, then washed with 1×PBS and processed for immunofluorescence. After 10 minutes of incubation in 50 mM ammonium chloride, cells were permeabilized 4 minutes with 0.1% Triton X-100, then incubated in 1×PBS. Hybridization of primary and secondary antibodies was performed in 1×PBS. Extracellular bacteria or InlB-beads were sometimes labeled prior to permeabilization. Vectashield hard set mounting medium with or without DAPI was applied (Vector Laboratories).

For incubation of host cells with bacteria, *L. monocytogenes* were grown as detailed above and added to cells at an MOI of 100. For kinetic analysis, cells were stored at 4°C for 5 minutes before incubation with bacteria, then once bacteria were added, cells were centrifuged at 1000 g for 1 minute at 4°C. Cells were then incubated at 37°C and 10% CO_2_ for 5, 10, and 15 minutes after which they were washed with 1×PBS, fixed with 4% PFA, and processed for immunofluorescence. To quantify the relative percentage of actin-positive *Listeria* during entry that also recruit septin, at least 200 actin-positive *Listeria* per cell line were analyzed at 5, 10, or 15 minutes post infection. Experiments involving cytochalasin D or nocodazole treatment were performed as described for other *Listeria* in vitro systems [51], [52], where cells were treated with DMSO, 5 µM of cytochalasin D, or 3.3 µM of nocodazole 30 minutes prior to infection. Kinetic analysis was performed as described above, though cells and bacteria were incubated in DMSO, cytochalasin D, or nocodazole throughout the infection. Cytochalasin D and nocodazole were suspended in DMSO and handled as suggested by the manufacturer (Sigma). For kinetic analysis of *S. flexneri* infection, infection of cells was performed as previously described [45]. Briefly, bacteria was grown in TCS as described above, and 400 µl of mid-exponential phase bacteria was centrifuged, resuspended in DMEM, and directly added to host cells. After centrifugation at 700 g for 10 minutes at 21°C, cells were then incubated at 37°C and 10% CO_2_ for 5 minutes. After these 15 minutes, cells were washed with 1×PBS, fixed with 4% PFA, and processed for immunofluorescence.

Images were acquired on a fluorescence inverted microscope Axiovert 200 M (Carl Zeiss MicroImaging, Inc.) equipped with a cooled digital charge-coupled device camera (Cool SNAP_HQ_, Photometrics) driven by Metamorph Imaging System software (Universal Imaging Corp). Confocal images were acquired using a Leica TCS SP5 (Leica microsystems). Immunofluorescence and confocal images were visualized and processed using the ImageJ public domain image processing software (http://rsb.info.nih.gov/ij/). Real-time immunofluorescence microscopy was performed using actin-YFP (as constructed in [36], BUG 2016) and SEPT9-CFP (the plasmid encoding the alpha variant of SEPT9 was a gift from S.E. Hilary Russell, BUG 2309). For this, JEG-3 cells were transfected using jetPEI (PolyPlus Transfection) according to the manufacturer's instructions with actin-YFP and SEPT9-CFP for 24 hours, and were infected with *L. monocytogenes* 1641. Infected cells were centrifuged at 1000 g for 1 minute at 4°C, and immediately placed on the microscope stage at 37°C. Image series were collected every 15 seconds for 15 minutes.

### RNA interference

HeLa cells (0.8×10^5^) were plated in 6-well plates (TPP) and transfected the following day using oligofectamine (Invitrogen) following the manufacturer's instructions. Scramble sequence custom designed from septin sequence [(sense) AUAAGCGACGUCCGCGUGGtt and (antisense) CCACGCGGACGUCGCUUAUtt] was applied as our control throughout experimentation. Custom scramble sequence, as well as pre-designed siRNA for SEPT2 (ID#14709) were both from Ambion, and handled according to manufacturer's instructions. Cells were tested 72 hours after siRNA transfection.

### Scanning electron microscopy

For scanning electron microscopy analysis, samples (non-infected siRNA-treated HeLa cells and siRNA-treated HeLa cells incubated with InlB-coated beads) were prepared as described above, subsequently washed in 1×PBS, prefixed in 2.5% glutaraldehyde in 0.1 M cacodylate buffer for 30 minutes, and then rinsed in 0.2 M cacodylate buffer. After post-fixation in 1% osmium tetraoxide (in 0.2 M cacodylate buffer), samples were dehydrated in a series of ethanol concentrations. Specimens were critical-point dried using carbon dioxide, then coated with gold and examined/photographed with a JEOL JSM-6700F scanning electron microscope (http://www.pasteur.fr/recherche/imagopole/microscopie.html).

### Fluorescence resonance energy transfer (FRET)

As previously described by our laboratory [36], PI 3-kinase activation can be measured by the production of its lipid products PI(3,4)P_2_ and PI(3,4,5)P_3_ which recruit the serine/threonine kinase Akt from the cytosol to the membrane, with the Akt pleckstrin homology (PH) domain specifically interacting with PI(3,4)P_2_ and PI(3,4,5)P_3_ [53], [54]. The quantification of FRET between two coexpressed fluorescent chimeras of the Akt PH domain, YFP-AktPH and CFP-AktPH, can be used as readout of PI 3-kinase activity. Without stimulation, the fluorescent chimeras are too dispersed in the cytosol to undergo FRET. Upon PI 3-kinase activation, the fluorescent AktPH chimeras concentrate at the plasma membrane, consequently leading to an increase in the FRET signal.

FRET occurs when an excited donor fluorophore (i.e. CFP) is located in close proximity to a lower-energy acceptor fluorophore (i.e. YFP). The proximity of fluorophores within the YFP-AktPH/CFP-AktPH complex allowed FRET stoichiometry measurements to quantify FRET inside cells. Image acquisition and processing for FRET stoichiometry were performed as originally described [55] and previously used in our laboratory [36]. In brief, siRNA-treated HeLa cells were co-transfected using jetPEI (PolyPlus Transfection) according to the manufacturer's instructions with plasmids encoding YFP-AktPH and CFP-AktPH. FRET stoichiometry measurement between YFP-AktPH and CFP-AktPH was applied as a readout of PI 3-kinase activation. Fluorescence images, corresponding to CFP, YFP, and FRET, and a fourth phase-contrast image were acquired every 15 seconds for 15 minutes with a consistent exposure time. A final concentration of 5 nM soluble InlB was supplemented after the 15^th^ frame. Application of the FRET stoichiometry equations, pixel by pixel, to the CFP, YFP, and FRET images was used to create a new image in which each pixel corresponds to (*E_A_*+*E_D_*)/2, a correction for variability in fluorescent chimera expression within and among cells [55], [56]. This term, referred to as FRET efficiency, is calculated by averaging the acceptor FRET efficiency (*E_A_*) with the donor FRET efficiency (*E_D_*), and allows comparison of FRET signals from cells expressing different molar ratios of fluorescent proteins. For kinetic studies we calculated, for each cell and time point, the mean (*E_A_*+*E_D_*)/2 over the entire cell. Final kinetics were obtained for 19 and 18 experiments, representing at least 3 biological replicates for each siRNA treatment, for control and SEPT2 siRNA-treated cells respectively.

To automatically detect whether a cell could be classified as having responded or not to InlB stimulation, we designed our own analysis software carried out using an Excel Macro. Our analysis software is based on these following criteria. From the original kinetic signals [i.e. the mean (*E_A_*+*E_D_*)/2 over the entire cell], a first median linear filter [formula: y_i_ = (∑_i−n/2,i+n/2_ y_i_)/n, n = 3] was applied to smooth artifact arising from the acquisition of original signal. The slope of each filtered curve was subsequently calculated for each time point, and was plotted as the processed ‘FRET response’ (i.e. as in Figure 5B). Starting from T_0_ (here defined as the time frame immediately following InlB stimulation, frame 16), T_max_ was detected when the slope again reached a minimum, where minimum slope is based on a null second derivative function. T_max_−T_0_ was used to define the duration of FRET response (where units are frames per 15 seconds). Between these 2 time points, the amplitude of the original signal [i.e. the mean (*E_A_*+*E_D_*)/2 over the entire cell] was calculated as a characteristic to qualify the response. As a final characteristic, the maximum slope was calculated between T_0_ and T_max_. This value also served to determine if the FRET response of that cell was positive. In the case where T_max_ could not be detected based on these criteria (i.e. the SEPT2-depleted cell from Figure 5B), such cells were then classified as not responding to InlB stimulation.

## Supporting Information

Movie S1 Real-time SEPT9-CFP recruitment at the site of Listeria entry in JEG-3 cells. Videomicroscopy was performed to demonstrate the recruitment of SEPT9-CFP collars at the site of Listeria entry in JEG-3 cells, while actin-YFP is shown to be recruited as a positive control. JEG-3 cells transfected with SEPT9-CFP and actin-YFP were infected with L. monocytogenes 1641, and were immediately placed on the microscope stage at 37°C. Image series were collected every 15 seconds for 15 minutes. The white arrow is pointing to the site of septin recruitment for the bacteria in the process of entering the transfected JEG-3 cell.
(3.08 MB AVI)

Movie S2 Representative FRET movie of a control siRNA-treated cell. HeLa cells treated with control (CTRL) siRNA were transfected with YFP-AktPH and CFP-AktPH. Transfected siRNA-treated cells were placed on the microscope stage at 37°C and image series were collected every 15 seconds for 15 minutes. Purified InlB was added to a final concentration of 5 nM after frame 15 (i.e. time point just prior to 4 minutes) after the start of imaging. A movie for the entire timecourse of InlB stimulation for the control cell depicted in Figure 5A is here presented (i.e. frame 0 to 60), showing the collected images (phase-contrast, Akt recruitment, and FRET efficiency). Pseudocolor scale represents the range of FRET efficiency values from original signal ranging from blue (low) to red (high).
(9.54 MB MOV)

Movie S3 Representative FRET movie of a SEPT2-depleted cell. HeLa cells treated with siRNA targeted against SEPT2 were transfected with YFP-AktPH and CFP-AktPH. Transfected siRNA-treated cells were placed on the microscope stage at 37°C and image series were collected every 15 seconds for 15 minutes. Purified InlB was added to a final concentration of 5 nM after frame 15 (i.e. time point just prior to 4 minutes) after the start of imaging. A movie for the entire timecourse of InlB stimulation for the SEPT2-depleted cell depicted in Figure 5A is here presented (i.e. frame 0 to 60), showing the collected images (phase-contrast, Akt recruitment, and FRET efficiency). Pseudocolor scale represents the range of FRET efficiency values from original signal ranging from blue (low) to red (high).
(6.84 MB MOV)
